# Professor Andreas Graner: driven by the quest to unlock crop plant genomes for conservation and utilization of germplasm for breeding

**DOI:** 10.1111/pbi.14143

**Published:** 2023-08-07

**Authors:** Rajeev K. Varshney, Nils Stein, Jochen Reif

**Affiliations:** ^1^ WA State Agricultural Biotechnology Centre, Centre for Crop and Food Innovation Murdoch University Murdoch WA Australia; ^2^ Leibniz Institute of Plant Genetics and Crop Plant Research (IPK) Gatersleben Seeland Germany

**Keywords:** genomics, genebank, genetic resources, genomics‐assisted breeding, barley, phenomics

## Abstract

Professor Andreas Graner stands as a towering figure in international crop plant genomics research, leaving an indelible imprint on the field over the past four decades. As we commemorate the 80th anniversary of Leibniz Institute of Plant Genetics and Crop Plant Research (IPK), Gatersleben, Germany and Professor Graner's retirement in September 2023, here we celebrate and acknowledge his profound impact on crop genome analyses and genebank genomics. His trailblazing work extends from developing the first integrated RFLP map of barley, establishing the foundation of barley genome sequencing, and advancing functional genomics of malting quality, to pioneering the use of high‐throughput phenomics. As the dedicated custodian of Germany's largest *ex situ* genebank at IPK Gatersleben, Professor Graner has fortified the institution's collection management and crop research, thereby contributing significantly to global efforts on conservation and utilization of plant genetic resources through genomics approaches. Alongside his impressive array of scientific achievements, Professor Graner's inspiring mentorship has nurtured a new generation of scientists, including us, leaving a lasting legacy in the field. This tribute underscores his enduring influence and celebrates his unwavering dedication to the scientific community.

## Introduction

**Figure 1:**
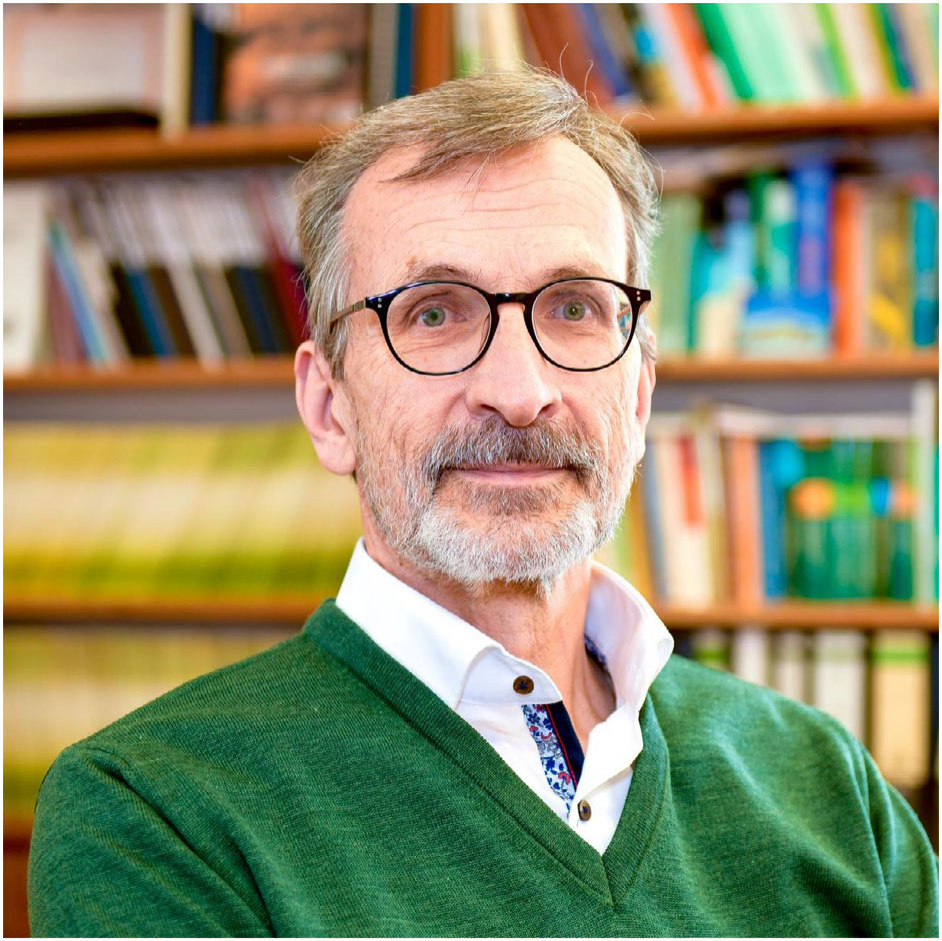
Professor Andreas Graner in his office at IPK, Gatersleben, Germany in the year 2023.

Embarking on an academic journey that transcends traditional disciplinary boundaries while maintaining a core focus can be a challenging endeavour. Yet, this is precisely the path that Professor Andreas Graner (Box 1; Figure 1) has navigated with remarkable success. With a career rooted in plant genetics and breeding, he has ventured into the arenas of molecular genetics, genomics and genebank management making significant contributions in each field (Table 1). Coined by his upbringing in southern Germany, a sense of rootedness is echoed in his research approach, where he has consistently ventured into emerging areas of plant genetics and breeding while maintaining his foundational expertise.

Box 1 **Life Sketch.**

Born on 5 October 1957, in Heilbronn, Germany, Professor Andreas Graner stands as an eminent figure in the field of plant genomics research. He was brought up in Bavaria, where he completed his high school. His academic journey began at Georg August University, Göttingen, Germany, where he pursued studies in Agricultural Sciences. Later, he transitioned to the Technical University of Munich, Germany, to complete his diploma. In 1987, he earned his PhD from the Technical University of Munich, with distinction, based on ‘Methodological Investigations on the Detection of Potato Spindle Tuber Viroid (PSTV)’. After the completion of his doctorate, he immersed himself in advanced research, initially serving as a Postdoctoral Fellow and subsequently, as a Research Scientist at the Institute for Resistance Genetics, Grünbach. In 1997, Professor Graner accomplished his ‘Habilitation’ at the Technical University of Munich in Applied Genetics and Plant Breeding, further consolidating his standing within the academic and scientific community. Afterwards, he joined IPK Gatersleben as a group leader and coordinator of the newly established Plant Genome Resources Centre. In 1999, he was appointed as head of IPK's genebank department and as a Professor of Plant Genetic Resources at Martin Luther University, Halle‐Wittenberg, Germany. His unwavering dedication to plant genetics research and his impactful leadership led to his appointment as the Managing Director of IPK Gatersleben in 2007, a position he continues to hold to this day.

**Table 1:** Contributions of Professor Andreas Graner to different research areas

Sr. no.	Research area	Years	Aspects
1	Molecular genetics	1987 onwards	Development and use of molecular markers (SSR, RFLP, EST, STS, AFLP and SNPs) in barley, wheat, rye and rice Linkage mapping and GWAS for various traits in barley, wheat and rice Comparison of genetic maps and physical maps in barley, wheat and rice
2	Applied genomics	1993 onwards	Marker‐assisted selection for barley yellow mosaic virus resistance and barley mild mosaic virus resistance
3	*Ex situ* conservation	1999 onwards	Conserve agrobiodiversity and provide the research community with plant genetic resources Improve the conservation management of genetic resources Transform genebanks into bio‐digital resource centres
4	Structural and functional genomics	2005 onwards	Cloning and characterization of candidate gene(s) for barley yellow mosaic virus resistance, malting quality in barley, lipoxygenases in barley, and leaf rust resistance in barley Transcriptome analysis in barley
5	Sequencing cereal genomes	2006 onwards	Sequencing the barley genome
6	High‐throughput phenomics	2007 onwards	Use of precision phenotyping systems for non‐invasive observation under controlled environmental conditions
7	Genebank genomics	2019 onwards	Capture and catalogue the genetic diversity available in genebanks using next‐generation sequencing technologies Population scale analysis of diversity panels and development of chromosome‐level reference genomes

Beyond his individual research accomplishments, Professor Graner has played a pivotal role in elevating the profile of his institution, Leibniz Institute of Plant Genetics and Crop Plant Research (IPK), Gatersleben, Germany, to international prominence in plant genetics and genomics. His leadership has fostered an environment of scientific excellence and innovation, nurturing generations of scientists, and promoting a research culture that pushes boundaries and challenges conventions. Ranking among the pioneers of molecular mapping of barley, he spearheaded genomics research into plant genetic resources, commonly referred as ‘genebank genomics’. Professor Graner's research has consistently been at the forefront of scientific discovery. His work reflects his forward‐thinking approach, his dedication to the advancement of plant genetic resources and his inspiring research leadership.

This article explores the various stages of Professor Graner's scientific journey, highlighting his key research contributions and his transformative leadership in the field. As we celebrate the 80th Anniversary of IPK in the month of September 2023, this tribute is a gift, acknowledging his remarkable accomplishments and his enduring impact on the field of crop plant genetics and genomics. This article also illuminates the extraordinary journey of a scientist who has skilfully interwoven multiple disciplines, thus contributing significantly to our understanding of crop plant genetics, genomics and germplasm research.

## Research contributions

### Molecular map of barley

**Figure 2:**
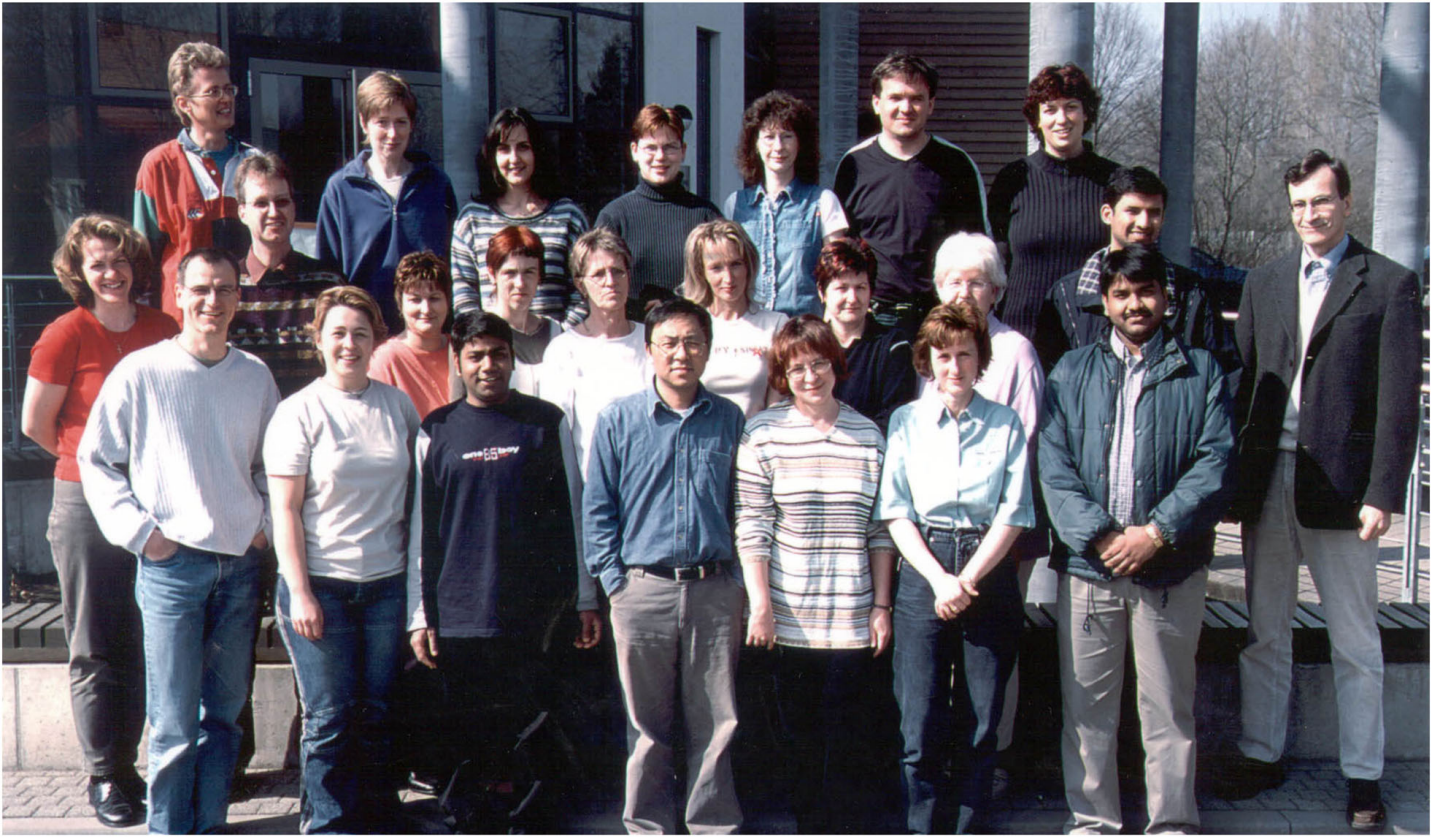
Professor Andreas Graner, along with his laboratory members at IPK Gatersleben, Germany, in the year 2003. Two authors of this article, Professor Nils Stein and Professor Rajeev K. Varshney, are standing in the front row in the first and the third place, respectively, and Professor Graner is standing on the outer right in the photograph.

Barley (*Hordeum vulgare*), an important cereal crop, holds the fourth position among grain cereals (Family *Poaceae*) globally, following maize, wheat and rice in terms of production. Despite having a large genome size of less than 5 Gbp, barley is considered an excellent genetic model for studying the genomes of large and complex genome crops like wheat owing to its relatively simpler diploid genome structure (2*n* = 2*x* = 14). This has been facilitated by the advent of molecular marker maps that have revolutionized our understanding of the structural characteristics of the barley genome. These maps encompass a wide array of selectable markers, which are instrumental in mapping various qualitative and quantitative traits within the genome. Some of these closely linked markers are routinely employed in marker‐assisted breeding programmes. However, this was not the case during the late 20th century when genetic marker mapping in barley posed challenges due to technical issues arising from the large genomes shared by most Gramineae species, which also contained extensive amounts of repetitive DNA. Additionally, the limited degree of polymorphism added to the complexities. Nonetheless, Professor Graner (Figure 2) played a crucial role in overcoming these obstacles by leading a team effort to construct a comprehensive RFLP (restriction fragment length polymorphism) map of the barley genome comprising over 200 RFLP probes. Graner *et al*. (1991) pursued two strategies to develop an RFLP map: (i) they utilized a population of 71 anther‐derived doubled haploid plants from an intraspecific cross to construct the first barley genetic map, and (ii) they analysed a population of 135 F_2_ individuals from a cross between cultivated (*Hordeum vulgare*) and wild barley (*Hordeum spontaneum*) to enrich the marker density. This RFLP map served as a crucial starting point for researchers across the globe for the genetic analysis of agronomic traits. Moreover, it facilitated further exploration of the barley genome at the molecular level.

### Mapping and characterization of resistance genes in barley

The primary challenge in applied genetics and genomics involves establishing a connection between genetic and phenotypic data and subsequently identifying the specific genes responsible for a particular trait. Since most traits are primarily determined by their observable characteristics rather than proteins or metabolites, the preferred approach for isolating genes of interest has been map‐based cloning. One example of such a trait is resistance to soilborne viruses in barley, such as barley yellow mosaic virus (BaYMV) and barley mild mosaic virus (BaMMV). These viruses are significant threats to winter barley production as they cannot be controlled chemically. The resistance of winter barley to BaYMV and BaMMV is predominantly governed by single recessive genes such as *rym4*, which provides complete immunity. In 1993, Professor Graner demonstrated one of the initial applications of marker‐assisted selection in barley breeding (Graner and Bauer, 1993). They employed a comprehensive RFLP map of the barley genome to precisely locate the *rym4* gene on the long arm of chromosome 3H. Additionally, they characterized the closely linked RFLP marker (MWG10) for use in marker‐assisted selection.

Subsequently, more resistance genes against BaYMV disease and a variety of fungal pathogens including *Phyrenophora teres*, *Rhynchosporium secalis* and *Puccinia hordei* were mapped in the following years by Professor Graner's group. Regarding virus resistance, the genetic architecture of the *rym5* locus was investigated using a progeny of 391 doubled haploid lines, which were evaluated for resistance against BaYMV‐1, BaYMV‐2 and BaMMV (Graner *et al*., 1999). While the presence of the *rym5* gene(s) conferred complete immunity, a novel gene called *rmm7*, providing partial resistance to BaMMV, was mapped on chromosome 1H. Furthermore, to facilitate positional cloning of the Rym4/Rym5 locus, two high‐resolution maps were created employing co‐dominant flanking markers (MWG838/Y57c10–MWG010/Bmac29; Pellio *et al*., 2005). The closely linked markers flanking the *rym4* and *rym5* genes held promise for marker‐assisted selection for virus resistance. Moreover, map‐based cloning of the rym4/5‐based recessive BaYMV resistance in barley led to the identification of the ‘eukaryotic translation initiation factor 4E’ (Hv‐eIF4E), one of the first resistance genes isolated and characterized in barley (Stein *et al*., 2005). Furthermore, the identification of SNPs specific to *rym4* and *rym5* revealed that these two alleles provide resistance against different variants of the virus. Consequently, the discovery of two alleles with distinct resistance specificities paved the way for leveraging the natural diversity present in *ex situ* or *in situ* collections to identify additional alleles conferring broad‐spectrum resistance to BaYMV (Yang *et al*., 2017).

### Advancement of the IPK genebank

Plant genetic resources play a crucial role in the study and utilization of biological diversity. Professor Graner, as the head of the federal *ex situ* genebank at the IPK in Gatersleben, Germany, bears the responsibility for one of the largest genebanks worldwide dedicated to agricultural and horticultural crop plants (https://www.ipk‐gatersleben.de/en/research/genebank). This genebank maintains collections at three sites, namely Gatersleben, Groß Lüsewitz and Malchow, comprising over 150 000 accessions from around the globe, significantly contributing to safeguarding cultivated plants and their wild relatives from extinction. Thanks to his unswerving efforts to converge the interests of two national Ministries, namely the Federal Ministry of Education and Research (BMBF), Federal Ministry of Food and Agriculture (BMEL) and the governments of three states, namely Saxony‐Anhalt, Saxony and Mecklenburg‐Vorpommern, he succeeded in unifying *ex situ* conservation in Germany under the leadership of IPK. In the course of this reorganization, the IPK genebank has been equipped with a new, efficient and comprehensive genebank database management system (Genebank Information System GBIS) in 2003. In subsequent years, the IPK genebank has further strengthened its international recognition as a pioneering institution in the preservation and quality management (QM) of plant germplasm. Notably, in 2007, IPK genebank achieved the distinction of becoming the second genebank globally to implement a QM system in compliance with ISO 9001 standards. Furthermore, in 2013, Professor Graner initiated the digitalization and consolidation of legacy data from the genebank, an important step towards bridging the gap between the genotype and phenotype.

**Figure 3:**
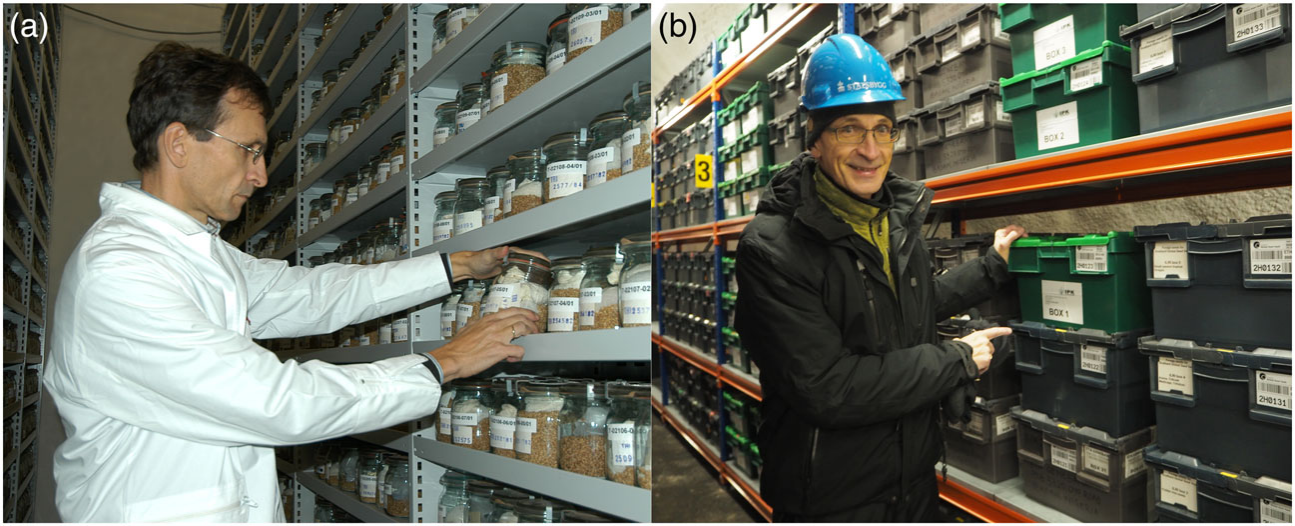
Lifelong dedication to plant genetic resources (a) Professor Graner inspecting crop genetic resources in the cold store of the *ex situ* genebank at IPK, Gatersleben, in 2004, and (b) depositing germplasm samples from IPK in the Global Seed Vault, Svalbard, Norway, in 2015.

Professor Graner has also played a significant role in improving the conservation management of genetic resources (see Figure 3). This was inspired by his vision of the genebank becoming an infrastructure promoting both research on and with plant genetic resources. To achieve this, the genomes of crucial crop species such as wheat, barley and rye are being extensively analysed. The focus lies on understanding the molecular basis of evolution and speciation, as well as identifying the drivers of intraspecific diversity. This effort aligns with the genebank's ongoing expansion of digital information services.

Professor Graner's contributions extend to seeking innovative solutions in scientifically based crop plant germplasm conservation and utilization, with the goal of improving resource efficiency and enhancing the sustainability of plant‐based production. By actively engaging with the DivSeek International Network, Professor Graner (as a Member of the Board of Directors, 2015–2021) has been playing a crucial role in transforming the IPK genebank from a mere ‘storage facility’ into a Bio‐Digital Resource Center (Mascher *et al*., 2019). It aims to become a comprehensive hub that facilitates and promotes the informed utilization of crop plant biodiversity. A key aspect involves leveraging the genomic data generated for the IPK germplasm collection by establishing an innovative and user‐friendly diversity informatics and data warehouse infrastructure capable of handling large‐scale datasets. This IT platform will establish links to the phenotypic legacy data accumulated in the genebank over six decades of conservation management. By facilitating access to such data, it creates a value chain that not only improves conservation management but also enhances the informed evaluation and utilization of crop genetic diversity for research and plant breeding purposes. This collective effort is helping to bridge the gap between genome information and the educated utilization of genetic diversity housed within the genebank.

### Sequencing the barley genome

**Figure 4:**
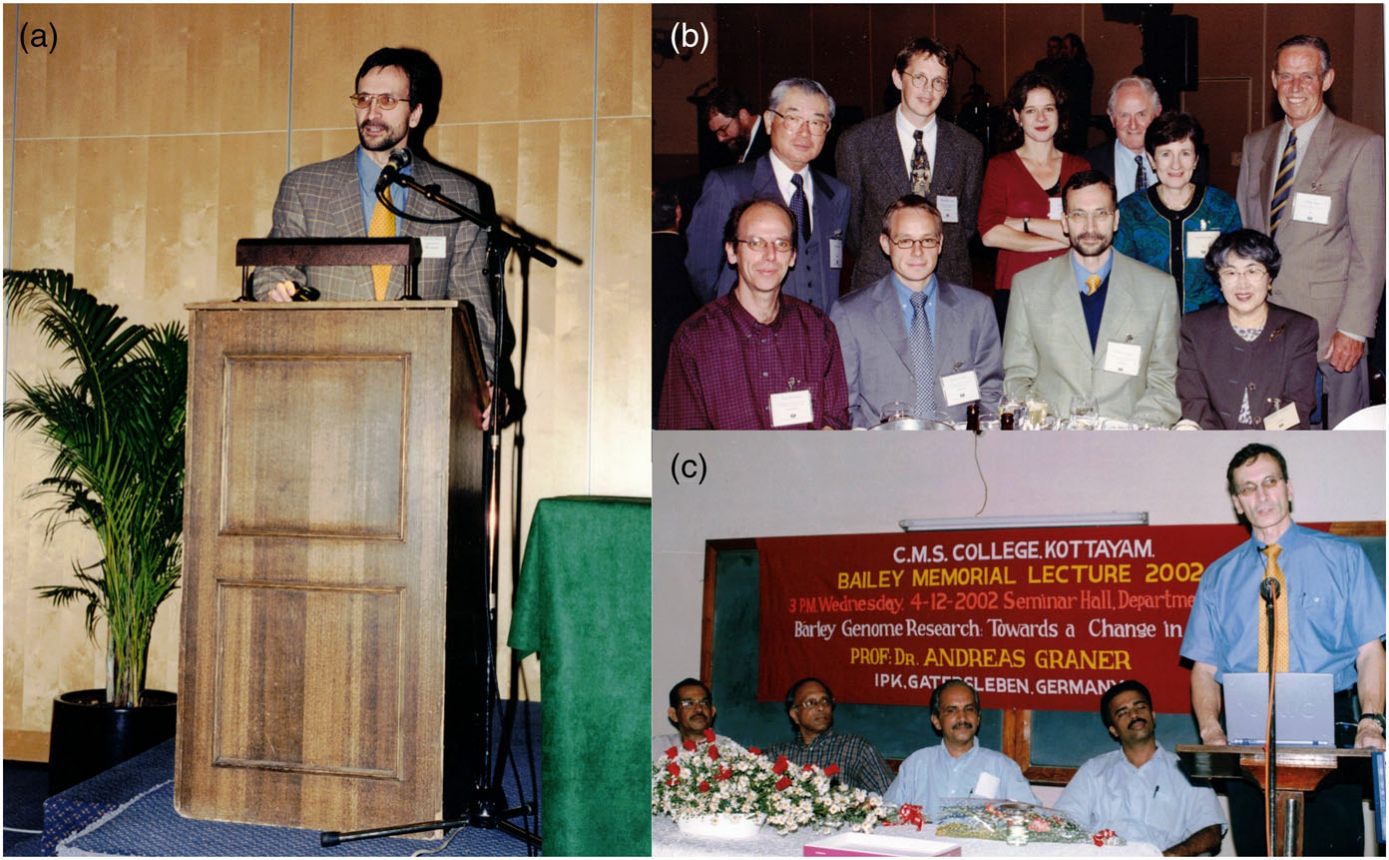
Notable appearances and engagements of Professor Andreas Graner during different meetings/conferences. (a) Professor Graner delivering a talk at the AgroGene meeting in Paris in 1998. (b) Professor Graner, along with several eminent scientists, during the International Barley Genetics Symposium at Adelaide, Australia, in 2000. (c) Professor Graner delivering a Bailey memorial lecture on ‘Barley Genome Research’ at C.M.S. College, Kottayam, India, in 2002.

With his exemplary vision and leadership, Professor Graner laid the cornerstone for sequencing the first barley reference genome. His role was instrumental in bringing together various research groups from four continents, fostering collaboration and establishing the International Barley Sequencing Consortium (IBSC) in the year 2006, which he chaired in its first years (Figure 4). As a leader of several projects funded under Genome Analysis of the Plant Biological System (GABI), a flagship programme of the Federal Ministry of Education and Research, BMBF, he contributed to the development of basic resources including the large‐scale generation and analysis of expressed sequence tags (Zhang *et al*., 2004), genic SSR, a generic *in silico* approach to developing SSR makers (Thiel *et al*., 2003; Varshney *et al*., 2005a), comprehensive genetic and transcript maps (Kota *et al*., 2007; Stein *et al*., 2007; Varshney *et al*., 2007). Subsequently, his mentee, Professor Nils Stein, spearheaded the efforts from IPK Gatersleben. IBSC developed a physical map of barley cultivar (cv.) Morex, which encompassed 4.98 Gb, with more than 3.90 Gb anchored to a high‐resolution genetic map. Approximately 84% of the assembled sequence consisted of repeat elements and harboured 26 159 ‘high‐confidence’ genes (IBSC, 2012). The completion of the barley reference genome has since been a vital resource for researchers worldwide, enabling a plethora of new discoveries and applications in crop improvement and sustainable agricultural practice.

### Pioneering functional genomics of malting quality

Professor Graner's innovative approach to bridge the gap between gene expression variations and phenotypic traits represents a major stride in barley functional genomics. His research targeted the complex trait of ‘malting quality’ in barley, utilizing a set of 10 diverse genotypes and analysing gene expression profiles during grain germination. The correlation of dissimilarity matrices, based on trait variation and gene expression data, enabled the identification of genes positively contributing to the observed differences in malting quality. This iterative procedure led to the identification of between 17 and 30 candidate genes for each of the six malting parameters analysed, thereby highlighting a promising strategy to connect functional genomics with plant breeding (Potokina *et al*., 2004).

### The dawn of a new era for genebank genomics

As a pioneering figure at the intersection of genomics and genebank management, Professor Graner has made seminal contributions to the understanding and utilization of plant genetic resources. His innovative use of molecular genetic strategies and forward‐thinking approaches have been instrumental in unlocking the potential of these resources. He has been involved in various key studies that epitomize the convergence of genebank and genomics (Mascher *et al*., 2019). He is considered one of the founding fathers of ‘genebank genomics’. One such study was a comprehensive analysis of genome‐wide genotyping‐by‐sequencing data for nearly all barley accessions (>20 K) of the German *ex situ* genebank. This ground‐breaking study provided crucial insights into the global population structure of cultivated barley, identified redundancies and gaps in the genebank, and pinpointed both known and novel loci underlying barley's morphological traits. The study laid a foundation for significantly enhancing germplasm management and underscoring the critical role of genomics in the effective utilization of genomic resources (Milner *et al*., 2019).

## Inspiring research leadership in action

The advice of Professor Andreas Graner was sought after by numerous institutions, scientific societies and committees. He served as the President of the German Society for Plant Breeding (GPZ) from 2016 to 2020. Additionally, he contributed his expertise as a member of the Scientific Coordination Committee of the BMBF Research Programme—GABI from 2000 to 2007. He also served on the Advisory Boards of several research institutes including the Max Planck Institute for Plant Breeding Research in Germany, the Otto Warburg Minerva Centre at The Hebrew University of Jerusalem in Israel, and the Julius Kühn Institute in Germany. Furthermore, he served on the Supervisory Board of the German Collection of Microorganisms and Cell Cultures (DSMZ), Germany, the CGIAR Generation Challenge Programme, c/o CIMMYT, Mexico, and the Steering Committee of the DivSeek International Network Inc., based in Canada. This global network serves as a platform for connecting, integrating and facilitating the exchange of knowledge among various stakeholders involved in the management and characterization of plant genetic resources. For more than 10 years, he served on the Advisory Board on Biodiversity and Genetic Resources of the Federal Ministry for Food and Agriculture (BMEL).

Before starting his career as a Senior Scientist, Professor Graner served as a Staff Scientist at the Federal Centre for Breeding Research on Cultivated Plants, Grünbach. In 1999, he was appointed the Head of the Federal *ex situ* genebank at IPK and Professor for Plant Genetic Resources at the University of Halle, Germany. Since 2007, Professor Graner has held the position of Managing Director at IPK, a research institute with more than 450 employees.

In addition to his leadership roles, Professor Graner has served as an editorial board member for renowned international scientific journals such as Euphytica from 1993 to 2005, Molecular Breeding since 2004, and Theoretical and Applied Genetics since 2006.

## Recognitions that illuminate a remarkable journey

**Figure 5:**
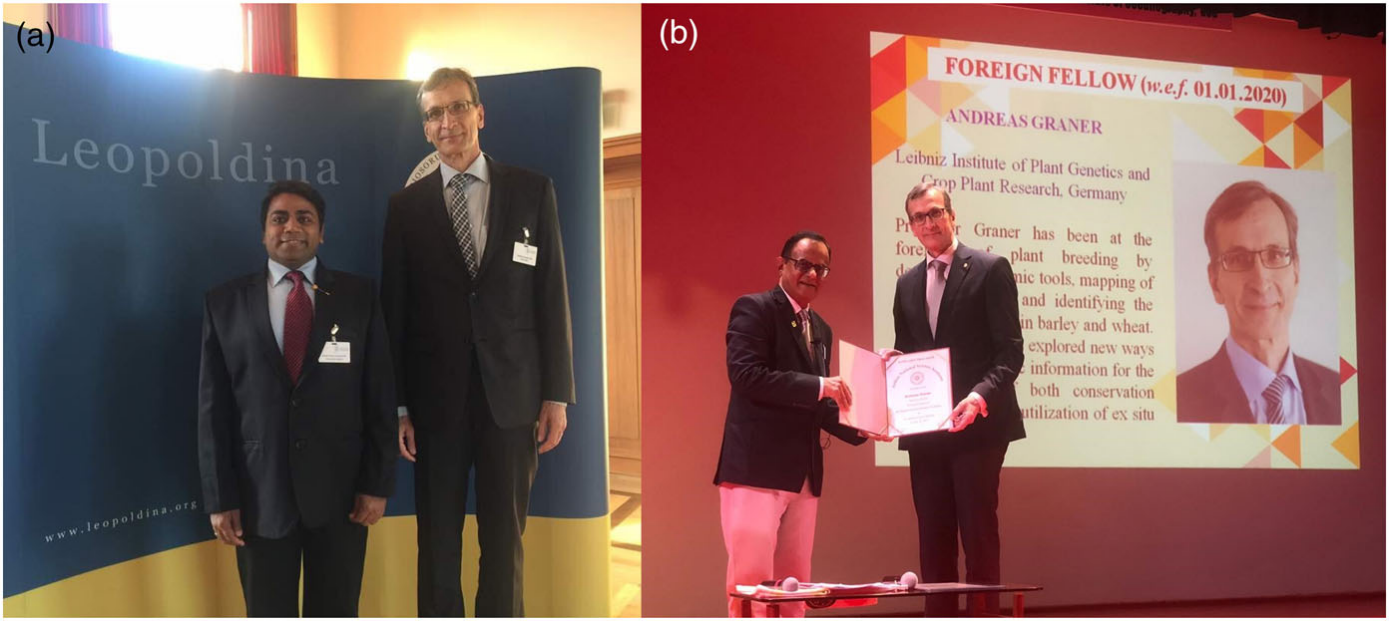
Professor Andreas Graner at National Science Academies of different countries. (a) Professor Graner with Professor Rajeev K. Varshney at the German National Academy of Sciences, LEOPOLDINA at Halle, Germany, in 2016. (b) Professor Graner receiving the INSA Fellowship award from Dr A. K. Sood FRS, President of the Indian National Science Academy (INSA) on being elected as the Foreign Fellow of the INSA in Goa, India, in 2020.

Over the course of his illustrious career, Professor Graner has been the recipient of numerous prestigious awards and honours and has been elected to several distinguished science academies **(**Figure 5; Table 2). His first significant recognition came in 1987 when he was bestowed with the ‘Kurt von Rümker Award’. In 2001, Professor Graner was elected as a member of the esteemed ‘German National Academy of Sciences, LEOPOLDINA’, a testimony to his profound contributions to global research. His work was further acknowledged in 2004 with the ‘Gregor Mendel Innovation Award’. In 2006, he was honoured as an Honorary Fellow at the ‘Scottish Crop Research Institute (SCRI)’, marking his international impact in the field. His research has consistently been influential and highly cited, earning him the Thomson Reuters Highly Cited Researcher award in 2015. Reflecting his growing global stature, in 2018, he became a member of the ‘National Academy of Agricultural Sciences (NAAS)’ in India, and in 2020, he was elected to the ‘Indian National Sciences Academy (INSA)’. Very recently, for his outstanding contributions and establishing a legacy in germplasm research, The Crop Trust (Germany) honoured him with its Legacy Award in 2023. These recognitions serve to underscore the enduring significance and broad impact of Professor Graner's work in plant genomics research.

**Table 2:** Professor Andreas Graner's accolades, recognition and organizational proficiency

Year	Accolades, recognition and organizational proficiency
2020–Present	Deputy chair, section agricultural and food science, LEOPOLDINA
2013–Present	Member, Scientific Advisory Board, Biodiversity and Genetic Resources, Federal Ministry of Food and Agriculture (BMEL), Germany
2008–Present	Member, Scientific Advisory Board, Julius Kuehn Institute (JKI), Quedlinburg, Germany
2013–Present	Member, Governing body, BGI Biotechpark Gatersleben Infrastrukturgesellschaft mbH
2014–Present	Chair, Working group, ‘Genetic Resources’ German Society for Plant Breeding (GPZ)
2007–Present	Member, Board of trustees, Charitable Foundation, Salzlandsparkasse
2007–Present	Chair, Society for the promotion of the School Laboratory Gatersleben, Germany
2006–Present	Editor, Theoretical and Applied Genetics, Springer, Germany
2004–Present	Editor, Molecular Breeding, Springer, The Netherlands
1999–Present	Vice Chair, Association for the Promotion of Crop Plant Research
2023	Crop Trust's Legacy Award
2016–2021	President, German Society for Plant Breeding (GPZ), Quedlinburg, Germany
2016–2021	Board of Trustees, Gregor Mendel Foundation, Bonn, Germany
2015–2021	Steering Committee, DivSeek Initiative
2020	Elected member, Indian National Science Academy (INSA)
2019–2020	President, Rotary Club Quedlinburg, Germany
2018	Elected member, National Academy of Agricultural Sciences (NAAS), India
2005–2016	Member, Scientific Advisory Board, Otto Warburg Centre, Hebrew University, Rehovot, Israel
2015	Thomson Reuters Highly Cited Researcher
2008–2015	Member, Executive Board, CGIAR Generation Challenge Programme, c/o CIMMYT, El Batan, Mexico
2006	Honorary Fellow, Scottish Crop Research Institute (SCRI)
2004	Gregor Mendel Innovation Award
2003–2011	Member, Scientific Advisory Board, Max Planck Institute for Plant Breeding Research Cologne, Germany (2009–2011 Chair)
2001	Elected member, German Academy of Sciences, LEOPOLDINA
1993–2006	Editor, Euphytica, Springer, The Netherlands
1987	Kurt von Rümker Award, German Society of Plant Breeding (GPZ)

## Summary

As reflected above, the professional life of Professor Graner has been driven by his quest to unlock crop plant genomes for the valorization of germplasm collections with an overall objective of efficient conservation and utilization of germplasm during the last four decades. He and his group coined several new concepts, including ‘genic SSR markers’ (Varshney *et al*., 2005a) and ‘genomics‐assisted breeding’ (Varshney *et al*., 2005b). Several of his mentees are leading various research and leadership positions in Germany, Australia, India and many other countries. In brief, his life journey is truly inspiring for the young generation and is expected to motivate researchers from all over the world to take up their research careers and make an impact in the area of crop genomics, genebank genomics and genomics‐assisted breeding.

## Conflict of interest

The authors declare no competing interests.
